# Contrasting effects of land‐use changes on herbivory and pollination networks

**DOI:** 10.1002/ece3.5814

**Published:** 2019-11-20

**Authors:** Naoto Shinohara, Kei Uchida, Takehito Yoshida

**Affiliations:** ^1^ Department of Agricultural and Life Sciences The University of Tokyo Tokyo Japan; ^2^ Institute for Sustainable Agro‐ecosystem Services The University of Tokyo Tokyo Japan; ^3^ Department of General Systems Studies The University of Tokyo Tokyo Japan; ^4^ Research Institute for Humanity and Nature Kyoto Japan

**Keywords:** agroecosystems, biodiversity, herbivore, interaction network, pollination, seminatural grassland

## Abstract

1. Land‐use changes, one of the greatest threats to global biodiversity, can cause underappreciated effects on ecosystems by altering the structures of interspecific interaction networks. These effects have typically been explored by evaluating interaction networks composed of a single type of interaction. Therefore, it remains unclear whether the different types of interaction networks sharing the same species respond to the same land‐use changes in a similar manner.
2. To compare the responses of herbivory and pollination networks to land‐use changes, we investigated both types of interaction networks in seminatural grasslands categorized into three types of agricultural land‐use (abandoned, extensively managed, and intensively managed) in a Japanese agricultural landscape. We quantified the structures of the interaction networks using several indices (connectance, evenness, diversity, generality, network specialization, and robustness) and compared them among different land‐use types. We conducted piecewise *SEM* to differentiate the direct and indirect effects of land‐use changes on the network structures.
3. Although both land‐use changes (abandonment and intensification) led to reduced plant and insect species richness, the structures of herbivory and pollination networks showed different responses to the land‐use changes. There was a marked contrast in network generality; while, herbivore species were less generalized (i.e., having fewer host plant species) in fields with land‐use intensification, pollinator species were less generalized in abandoned fields.
4. Furthermore, the mechanisms behind the changes in interaction networks were also different between pollination and herbivory networks. The change in herbivory network generality was induced by the decrease in plant species richness, whereas the change in pollination network generality was mainly induced by the effect independent of changes in species richness and composition, which possibly reflect the less number of flowers in shaded environment.
5. The present study demonstrates that agricultural land‐use changes affect herbivory and pollination networks in contrasting ways and suggests the importance of assessing multiple types of interaction networks for biodiversity conservation in plant–insect systems. Our results also highlight the underappreciated importance of maintaining habitats with an intermediate intensity of land‐use.

## Graphical abstract

Land‐use changes had contrasting effects on herbivory and pollination networks, and the mechanisms behind the changes in the interaction networks were also different.

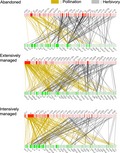

## 1 INTRODUCTION

Studies have consistently reported that anthropogenic land‐use changes modify terrestrial ecosystems (Foley et al., 2005; Pimm et al., 2014; Sala et al., 2000). While their effects have typically been reported in terms of declining species diversity (Billeter et al., 2008; Kleijn, Rundlöf, Scheper, Smith, & Tscharntke, 2011; Newbold et al., 2015), it is increasingly acknowledged that habitat loss and degradation induced by land‐use changes modify the structures of species interaction networks (Albrecht, Duelli, Schmid, & Müller, 2007; Tylianakis & Morris, 2017; Tylianakis, Tscharntke, & Lewis, 2007). The structures of such networks are vital to community dynamics and stability (Bascompte, Jordano, & Olesen, 2006; May, 1972; Thébault & Fontaine, 2010) and land‐use changes can modify network structures without affecting species richness (Tylianakis et al., 2007). Therefore, investigating the effects of land‐use changes on species interaction networks, as well as on species diversity, is one of the major conservation challenges (Tylianakis, Laliberté, Nielsen, & Bascompte, 2010).

However, most studies to date have focused on a single type of interaction (e.g., Albrecht et al., 2007; Lázaro et al., 2016) despite the multiple types of interactions involved in ecological communities (Montoya, Pimm, & Solé, 2006; Pocock, Evans, & Memmott, 2012). Therefore, it remains unclear whether different types of interaction networks respond to land‐use changes in a similar manner (but see Albrecht et al., 2014; Grass, Jauker, Steffan‐Dewenter, Tscharntke, & Jauker, 2018). The effects of land‐use changes are predicted to differ between different types of interaction networks (Grass et al., 2018) because antagonistic and mutualistic interaction networks exhibit different structures (Thébault & Fontaine, 2010) and trophic, mutualistic, and parasitic interaction networks sharing the same plant communities do not necessarily show the similar response to sequential species removal (Pocock et al., 2012). Therefore, focusing on a single type of interaction network could not help us understand the total effect of land‐use changes on ecological communities, including various taxonomic groups (Pocock et al., 2012).

In addition, because most studies have focused on the effects of land‐use intensification (e.g., agricultural intensification: Albrecht et al., 2007; Marrero, Torretta, & Medan, 2014; Weiner, Werner, Linsenmair, & Blüthgen, 2011, grazing: Lázaro et al., 2016; Vanbergen et al., 2014, and urbanization: Baldock et al., 2015; Theodorou et al., 2017), how interaction networks respond to the land‐use change toward the underuse, such as land‐use abandonment, remains poorly understood. As land‐use abandonment is thought to be as great a threat to plant and insect diversity as land‐use intensification (Koshida & Katayama, 2018; Middleton, 2013; Normile, 2016; Queiroz, Beilin, Folke, & Lindborg, 2014; Uchida & Ushimaru, 2014), land‐use abandonment is also expected to modify interaction networks between plants and insects. Importantly, Lázaro et al. (2016) found that pollination network complexity was highest at the intermediate level of the grazing gradient. Therefore, though abandonment and intensification may be the similar change of land‐use intensity (though with opposite directions), it is crucial to evaluate the bidirectional land‐use changes separately to detect the actual pattern such as a unimodal pattern along a full gradient of land‐use intensity.

Disentangling the mechanisms by which land‐use changes affect interaction networks is also an important challenge and remains a controversial topic. Due to the strong relationship between network structure and size (i.e., the number of species comprising the network; Bersier, Dixon, & Sugihara, 1999; Fründ, McCann, & Williams, 2016; Goldwasser & Roughgarden, 1997), it is often concluded that the effects of land‐use changes on interaction network structures are accounted for by the changes in the interaction network size (Baldock et al., 2015; Vanbergen et al., 2014). In contrast, several studies have suggested that interaction network structures are modified independently of changes in the network size (Lázaro et al., 2016; Tylianakis et al., 2007). In the latter case, the changes in interaction networks are expected to be accompanied by changes in species composition. Particularly, as specialist insect species are more vulnerable to the loss of host plant species than generalist species (Weiner, Werner, Linsenmair, & Blüthgen, 2014), changes in the generalist–specialist ratio are expected to be related to interaction network structures (de Araújo, Vieira, Lewinsohn, & Almeida‐Neto, 2015).

In this study, we studied two types of interaction networks between plant and insect species (herbivory and pollination) in seminatural grasslands formed in a Japanese agricultural landscape. This system was ideal for our study objective because the landscape is an ensemble of different land‐use types (Fukamachi, Oku, & Nakashizuka, 2001; Kadoya & Washitani, 2011), allowing us to observe the great variation between local communities within a limited area. We categorized the 12 seminatural grasslands into three classes of land‐use according to the level of intensity (abandoned, extensively managed, and intensively managed; in order from the least intense to the most intense) and explored the responses of the interaction networks to the bidirectional land‐use changes, namely abandonment (from extensively managed to abandoned fields) and intensification (from extensively managed to intensively managed fields). This study was designed to investigate whether the bidirectional land‐use changes affect different types of interaction networks similarly and whether the effects are caused by changes in species richness or species composition. Specifically, we tested the following hypotheses. (a) Both land‐use changes (intensification and abandonment) decrease the plant and insect species richness and change species composition, although (b) their effects on herbivory and pollination networks are different. Whereas intensification is expected to decrease the herbivory network complexity as suggested by a meta‐analysis (de Araújo et al., 2015), its effect on pollination networks might not be detected because the previous studies have reported various contrasting results (Lázaro et al., 2016; Nielse & Totland, 2014; Vanbergen et al., 2014). As for abandonment, pollination networks are expected to be less complex in abandoned fields (Lázaro et al., 2016), whereas we have no specific hypothesis about herbivory networks because of the lack of previous studies testing this. Furthermore, we hypothesized that (c) the effects of land‐use changes on the interaction networks are threefold. The effects can be mediated by changes in network size (Baldock et al., 2015; Vanbergen et al., 2014) or by those in species composition (e.g., the generalist–specialist ratio; de Araújo et al., 2015; Weiner et al., 2014). In addition, land‐use changes can affect network structures independently of network size (Lázaro et al., 2016; Tylianakis et al., 2007) and species composition (Theodorou et al., 2017), but by changing the traits of plants and insect species (e.g., flowering and diet preference).

## 2 MATERIALS AND METHODS

### 2.1 Study sites, land‐use types, and land‐use changes

**Figure 1:**
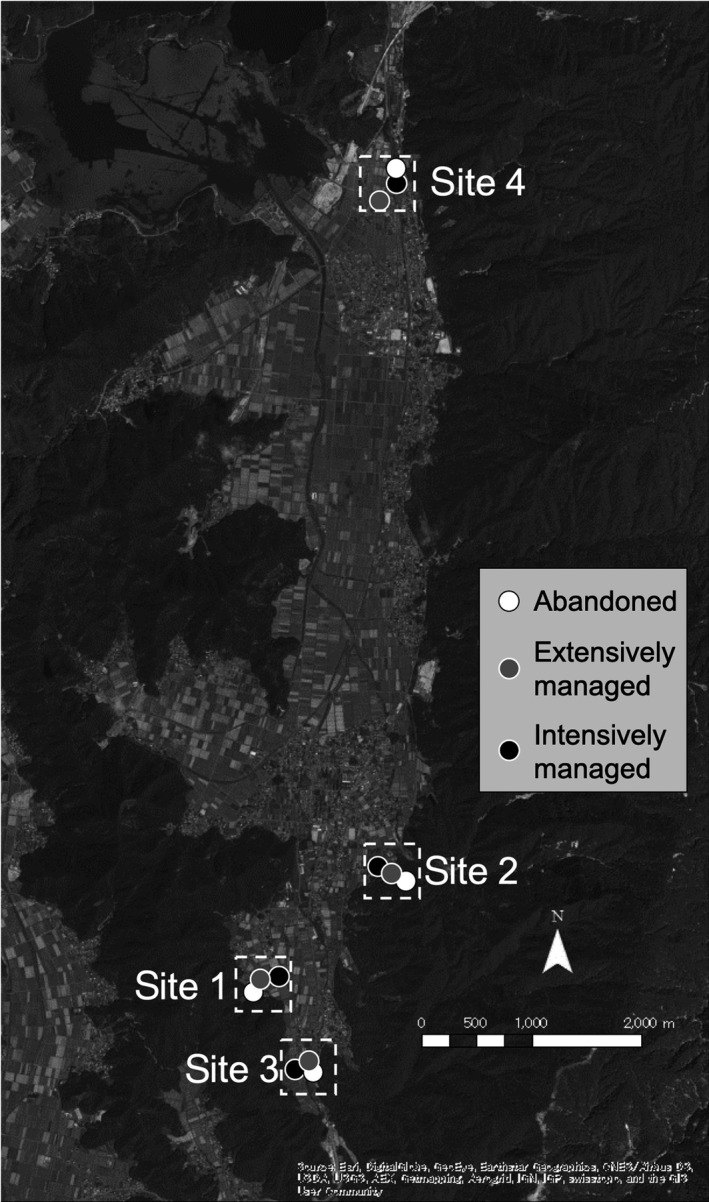
Location of the study sites and plots in Wakasa Town, Fukui Prefecture, Japan. Dashed rectangles and circles represent the sampling sites (500 m × 500 m) and the sampling plots (white, abandoned; gray, extensively managed; and black, intensively managed), respectively. The background aerial photo was taken by WV02 of DigitalGlobe on 31 August 2012

The study plots were located in an agricultural landscape dominated by rice fields (ca. 40 km^2^, 35°29′–33′N, 135°52′–54′E) in Wakasa town, Fukui Prefecture in central Japan (Figure 1). The mean annual temperature is 14.8°C, with a minimum monthly average temperature of 4.2°C in January and a maximum monthly average temperature of 26.7°C in August; the mean annual precipitation is 2,092.2 mm (averaged over 1981–2010; the data were obtained from the Japan Meteorological Agency). In Japanese agricultural areas, farmers periodically mow the edges of rice fields for the convenience of agricultural management, resulting in the maintenance of long and narrow seminatural grasslands (Figure S1). Although the seminatural grasslands on the edges of extensively managed fields are known to harbor a great number of plant species (Bambaradeniya et al., 2004; Fukamachi, Oku, & Miyake, 2005), many of the fields have been abandoned or highly intensified, both of which result in reduced plant and insect diversity (Kiritani, 2000; Koshida & Katayama, 2018; Uchida & Ushimaru, 2014).

We categorized the studied seminatural grasslands into three types of land‐use according to the level of land‐use intensity, as follows (Figure S1):

1. *Abandoned fields* were defined as former rice fields where farmers had ceased rice cultivation ≥10 years ago, and the fields and their edges had been no longer mowed.
2. *Extensively managed fields* were defined as rice fields that were being traditionally extensively managed by low‐frequency mowing (two or three times a year) of the edges. These fields had not been subjected to land consolidation into larger, more productive fields and remained relatively small and irregularly shaped.
3. *Intensively managed* fields were defined as rice fields that had experienced land consolidation twice (ca. 10 and 40 years ago) accompanied by the destruction and restoration of their edges. These fields are characterized by highly intensive management with a high frequency of mowing (four or more times a year) of their edges.

Four clusters of study plots were established in the study area to have the three land‐use types in each cluster (12 plots in total; the clusters are shown in Figure 1 and referred to as “sites” hereafter). To remove the possible confounding effect of topography associated with the land‐use types, the three study plots were selected to be located as near as possible to each other within each site (within 500 m, Figure 1). Given that insect species can move over a wide range (e.g., bees can fly up to several kilometers), insect communities within each cluster might not be considered as independent. To assess this, we performed permutational multivariate analyses of variance (perMANOVA), with the pollinator composition or herbivore composition in each plot as a response variable and season, land‐use types, and sites (clusters) as explanatory variables. Insect community composition was mainly explained by seasons (for pollinator, partial *R* ^2^ = .160, *p* = .001; for herbivore, partial *R* ^2^ = .130, *p* = .001) and land‐use types (for pollinator, partial *R* ^2^ = .172, *p* = .001; for herbivore, partial *R* ^2^ = .075, *p* = .009), but was explained little by sites (for pollinator, partial *R* ^2^ = .021, *p* = .500; for herbivore, partial *R* ^2^ = .032, *p* = .119). Therefore, insect communities were not clustered spatially at the site scale, and an insect community in each plot was more strongly influenced by local mechanisms rather than possible interplot dispersal.

In the following, the effects of land‐use changes (abandonment and intensification) were analyzed by comparing the response variables in extensively managed fields with those in abandoned or intensively managed fields, respectively. These comparisons reflect the past conversion of extensively managed fields which used to be common in Japanese agricultural areas, into abandoned fields due to a rapid decrease in the number of farmers (Fukamachi et al., 2005, 2001) or into intensively managed fields for improved productivity (Uematsu, Koga, Mitsuhashi, & Ushimaru, 2010).

### 2.2 Field survey

We studied herbivory and pollination interactions and plant and insect species composition in the 12 study plots in three seasons: spring (29 May–11 June 2016), summer (28 June–11 July 2016), and autumn (5 September–24 September 2016).

A transect (2 m × 30 m) was established for each plot. First, we simultaneously surveyed both herbivory and pollination interactions in each transect twice for each season. For each transect and season, the survey was performed in both the morning (8:00–10:30 a.m.) and the afternoon (12:30–15:00 pm), on the same day if the weather permits. Each survey included walking for 150 min along the transect while carefully and exhaustively observing insect species that exhibited either of herbivory behavior or floral visitation. The herbivory behavior was counted when we observed individual insects consuming leaves or stems of an individual plant species. Regarding floral visitation, we counted individual insects that touched plant reproductive parts. Due to the large number of observations of the interactions, we were not able to evaluate whether the insects actually carried pollen. Therefore, the interactions might not be mutualistic, and the insects are flower visitors rather than pollinators. However, since the most insect individuals were identified as the taxonomic groups known as common pollinators, we assume that most insects contribute to pollination to some degree and refer to them as pollinators. While many of the insect individuals were identified to species level (1,531 individuals), some individuals (263 individuals) could not be determined to species level and were therefore designated as morphospecies (32 morphospecies; Table S1).

Second, plant species composition was also investigated. We established five quadrats (50 cm × 50 cm) on the transect of each plot. For each quadrat, all the plant species present were recorded with their relative abundance and number of entomophilous flowers.

### 2.3 Network indices

For each plot and each season, by combining the data obtained from the surveys in the morning and the afternoon, we built herbivory and pollination interaction networks with their links reflecting the observation frequency, 72 interaction networks in total (two types of interaction, three types of land‐use, four sites, and three seasons). Among the various interaction network indices (Bersier, Banašek‐Richter, & Cattin, 2002), we used six commonly‐used quantitative indices to represent the network structure. “Connectance” represents the realized fraction of links in the network; “evenness” represents Shannon's evenness index for the network links; “diversity” represents Shannon's diversity index for the network links; “generality” represents the mean number of plant species for each insect species weighted by the interaction frequency; “H2′” represents network‐level specialization ranging from 0 (no specialization) to 1 (complete specialization; Blüthgen, Menzel, & Blüthgen, 2006); and “robustness” is the area under the secondary extinction curve from simulations of secondary extinctions of higher trophic level (herbivore or pollinator) species following the random sequential loss of plant species (see Memmott, Waser, & Price, 2004; Pocock et al., 2012). These indices were calculated using the “bipartite” package (Dormann, Fründ, Blüthgen, & Gruber, 2009) in R 3.3.1 (R Development Core Team, 2016).

Because less than two insect species were observed in three pollination networks, network indices including connectance, evenness, diversity, generality, and robustness were not calculated. Similarly, due to the limited number of plant and insect species in the network (less than two insect species or two plant species), H2′ was not calculated for five pollination networks and three herbivory networks. As our observation was relatively intense (5 hr per a network), we consider that these limited numbers of species were not due to the limitation of sampling efforts, but rather reflecting the actually small network size.

### 2.4 Statistical analyses

All the statistical analyses were conducted using R 3.3.1 (R Development Core Team, 2016). First, the effects of the land‐use changes on insect and plant species richness were evaluated using generalized linear models (GLMs). We constructed GLMs (Poisson error distribution and log link function) with land‐use type and study season as fixed variables. The response variables were the total number of plant species, flowering plant species, insect species, herbivore species, and pollinator species. The significance of the total effect of land‐use type was evaluated based on a likelihood ratio test (LRT). Afterward, we conducted pairwise comparison (extensively managed vs. abandoned or intensively managed) with GLMs to study the effect of land‐use changes (abandonment and intensification) as post‐hoc tests.

Second, the effects of land‐use changes on the network indices (connectance, evenness, Shannon's diversity, generality, H2′, and robustness) were also evaluated using GLMs with land‐use type and study season as fixed variables. We used Gamma distribution with inverse link function for H2′ because the normality of this response variable was not confirmed (Kolmogorov–Smirnov test; *p* < .05). For the rest of the indices, we used Gaussian error distribution and log link function after confirming the normality of their log‐transformed value (Kolmogorov–Smirnov test; *p* > .05).

Third, the plant (those recorded by plant composition surveys), herbivore, and pollinator species composition in different land‐use types were ordinated with nonmetric multidimensional scaling (NMDS; Anderson, 2001) with Bray–Curtis distances, using the “vegan” v.2.4‐4 package (Oksanen et al., 2018). The differences between the land‐use types were tested using permutational multivariate analysis of variance (perMANOVA) with Bray–Curtis distances, fitting land‐use types as an explanatory variable and season as a covariate variable.

Finally, to differentiate the total effect of land‐use changes from the effects mediated by network size, species composition, and the other independent effect, we conducted piecewise structural equation modeling (piecewise *SEM*) with the “piecewiseSEM” package (Lefcheck, 2016). We adopted this method rather than the network standardization method used in previous studies (Lázaro et al., 2016; Vanbergen et al., 2014) because it allows to specifically test the dependence relationships between variables (Lefcheck, 2016) and calculate the relative importance of different pathways (see Barnes et al., 2017; Grass et al., 2018). We modeled the effects of land‐use changes on network indices via changes in both network size (plant and insect species richness) and plant and insect species composition, as well as the other independent effect. As for insect species composition, we specifically hypothesized that the changes in network indices can be attributed to the generalist–specialist ratio in insect communities (de Araújo et al., 2015; Weiner et al., 2014). To test this hypothesis, we calculated “fundamental generality” for each insect species as the number of plant species it utilizes (as herbivory or pollination) in the all networks and incorporated its averaged value for the insect species within each network as the insect species composition variable. As the plant species composition variable, the first axis score of the NMDS was incorporated. In addition, we also hypothesized the covariance paths between species richness and composition for plants and insects, and causal paths from plant species richness and composition to insect species richness and composition. Each path was modeled as GLM (Gaussian error distribution and log or identity link function) with seasons as a covariate. To obtain the best model, we deleted nonsignificant paths in a stepwise manner by removing the path with the highest *p* value until only the paths with *p* < .1 remained. As the objective of this analysis was to investigate the effects of land‐use changes on network indices, we constructed piecewise *SEM*s only when we found the significant or marginally significant effect of land‐use changes on the indices (*p* < .1, see Table 2), which results in five separate piecewise *SEM*s (the effects of abandonment on pollination network connectance, diversity, and generality; the effects of intensification on pollination network connectance, and herbivory network generality). Although the nonsignificant effect masked by the opposing effects of different paths or the interdependence between the network indices are worth investigated, they are beyond our scope here. The fit of each piecewise *SEM* was tested using a Shipley's *d*‐separation test, and the model was regarded as fitted if *p* > .05.

## 3 RESULTS

### 3.1 Species richness and composition

A total of 129 plant species (90 in spring, 82 in summer, and 72 in autumn) and 166 insect species (74 in spring, 82 in summer, and 82 in autumn) were recorded.

The total and flowering plant species richness were significantly different between land‐use types (Table 1). Plant species richness was significantly higher in the extensively managed fields than in the abandoned (*z* = 6.54, *p* < .001; Table 1) and intensively managed fields (*z* = 2.51, *p* = .012), whereas species richness of flowering plants in the extensively managed fields was significantly higher than in the abandoned fields (*z* = 5.20, *p* < .001) but not the intensively managed fields (*z* = 1.64, *p* = .101).

**Table 1:** Species richness of plants and insects (herbivores and pollinators) in different land‐use types

	Abandoned (A)	Extensively managed (E)	Intensively managed (I)	LRT			Post‐hoc comparison (GLM)	
				*χ* ^2^	*df*	*p* value	A vs. E (*p*)	I vs. E (*p*)
Plant species richness								
Total	16.6 ± 5.7	28.4 ± 5.3	22.8 ± 4.9	90.0	2	**<.001**	**<.001**	**.012**
Flowering	1.4 ± 1.2	5.8 ± 3.6	4.3 ± 2.5	34.6	2	**<.001**	**<.001**	.101
Insect species richness								
Total	10.8 ± 2.7	14.3 ± 5.2	11.7 ± 3.5	6.00	2	**.049**	**.026**	**.053**
Herbivore	7.2 ± 2.3	6.3 ± 2.5	5.7 ± 2.7	2.93	2	.231	.432	.355
Pollinator	3.8 ± 3.1	8.1 ± 3.9	6.0 ± 2.7	17.4	2	**<.001**	**<.001**	**.076**
*N*	12	12	12					

Note

Significant *p* < .1 difference was shown in bold.

Values represent the means ± *SD*. The difference between land‐use types was tested by likelihood ratio test (LRT), and the statistics are shown. The post‐hoc pairwise comparison was conducted with GLMs and their significance levels are shown.

The total insect and pollinator species richness were significantly different between land‐use types, though herbivore species richness was not (Table 1). Total insect species richness in the extensively managed fields was significantly higher than in the abandoned fields (*z* = 2.24, *p* = .026) and the intensively managed fields (*z* = 1.94, *p* = .053). Among the insect species, pollinator species richness in the extensively managed fields was significantly higher than in the abandoned fields (*z* = 4.04, *p* < .001) and the intensively managed fields (*z* = 1.78, *p* = .076).

The NMDS ordination indicated that the compositions of plant, herbivore, and pollinator communities in the abandoned and extensively managed fields were significantly different (PERMANOVA: plant, *F* = 7.04, *p* < .001; herbivore, *F* = 1.62, *p* = .017; pollinator, *F* = 4.63, *p* = .001; Figure S2). In contrast, the community composition of the intensively managed and extensively managed fields was not significantly different (plant, *F* = 1.44, *p* = .107; herbivore, *F* = 0.803, *p* = .781; pollinator, *F* = 0.831, *p* = .60; Figure S2).

### 3.2 Interaction networks

**Figure 2:**
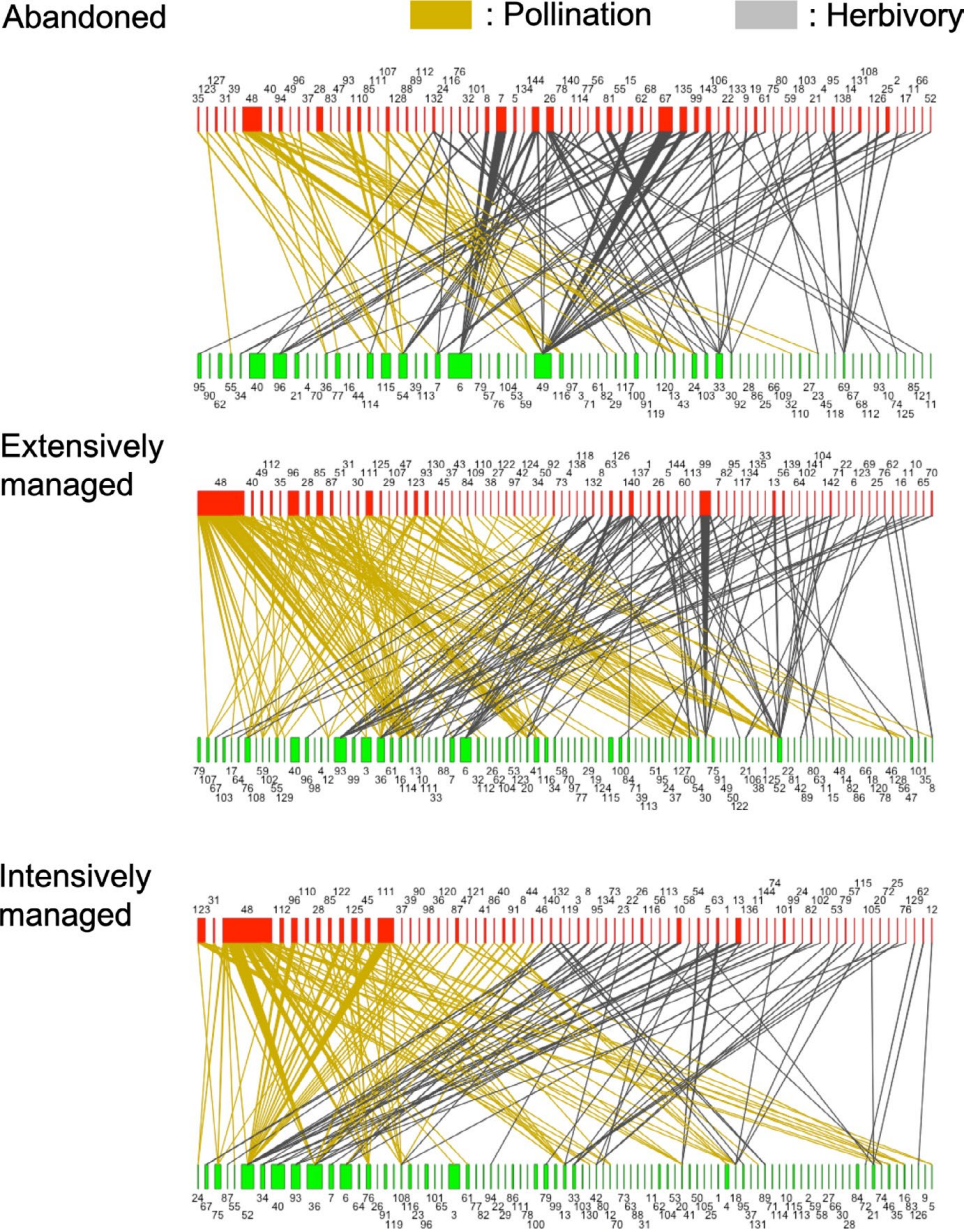
Herbivory and pollination networks observed in different land‐use types. For each network, the upper bars represent insect species with their relative frequency of interactions shown by the bar width, whereas the lower bars represent plant species with their relative abundance. The interaction networks shown here are based on the pooled observation data for all sites and seasons. Species IDs are given in Table S1

**Figure 3:**
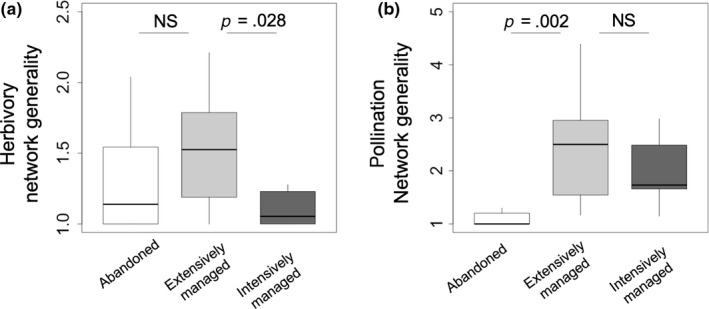
Contrasting effects of land‐use changes (abandonment and intensification) on the generality of (a) herbivory and (b) pollination networks. Boxes represent the median and 25th/75th percentiles and whiskers extend to 1.5 times the interquartile range. Comparisons were performed using GLMs and the significance levels of the differences (*p* values) are shown. NS, not significant

From the field survey (108 hr in total) yielding 1,802 observations of interactions between plant and insect species (609 for herbivory and 1,193 for pollination), 72 interaction networks were constructed (Figure 2 and Figure S2). Connectance, diversity, generality, and H2′ of pollination networks, and generality of herbivory networks were significantly different between land‐use types (Table 2). The effects of land‐use changes (abandonment and intensification) on the network indices were evaluated by pairwise comparison. Land‐use abandonment increased the connectance (*t* = 0.447, *p* = .010; Table 2) and decreased the diversity (*t* = −2.861, *p* = .011) and generality (*t* = −3.650, *p* = .00; Figure 3) of pollination networks. Land‐use intensification increased connectance of pollination networks (*t* = 2.339, *p* = .030) and decreased generality of herbivory networks (*t* = −2.361, *p* = .028; Figure 3).

**Table 2:** Summary of the effects of land‐use changes (abandonment and intensification) on the network indices (connectance, evenness, diversity, generality, H2′, and robustness) of herbivory and pollination networks

Network type	Network indices	LRT			Post‐hoc comparison (GLM)					
					Abandonment (Abandoned vs. extensively managed)			Intensification (Intensively vs. extensively managed)		
		*χ* ^2^	*df*	*p* value	Coefficient	*t* value	*p* value	Coefficient	*t* value	*p* value
Pollination	Connectance	**0.049**	**2**	**.006**	**0.447**	**2.899**	**.010**	**0.227**	**2.339**	**.030**
	Evenness	0.023	2	.375	0.085	0.988	.337	0.094	1.502	.149
	Diversity	**3.301**	**2**	**.003**	**−0.475**	**−2.861**	**.011**	−0.119	−1.126	.274
	Generality	**10.135**	**2**	**.000**	**−0.873**	**−3.650**	**.002**	−0.218	−1.431	.168
	H2′	**1.248**	**2**	**.069**	−0.941	−1.750	.101	−0.371	−0.906	.376
	Robustness	0.015	2	.411	0.073	1.097	.288	0.070	1.622	.121
Hebrivory	Connectance	0.003	2	.617	0.095	0.831	.416	0.136	0.864	.398
	Evenness	0.01	2	.369	0.046	0.824	.420	−0.032	−0.539	.596
	Diversity	1.089	2	.102	0.059	0.521	.608	−0.185	−1.513	.146
	Generality	**0.693**	**2**	**.056**	−0.142	−1.288	.213	**−0.251**	**−2.361**	**.028**
	H2′	0.178	2	.380	−0.126	−0.746	.465	−0.224	−1.264	.222
	Robustness	0.002	2	.729	0.010	0.242	.811	−0.024	−0.552	.587

Note

Significant *p* < .1 difference was shown in bold.

The overall difference between land‐use types was tested by likelihood ratio test (LRT), and the statistics are shown. The post‐hoc pairwise comparison was performed using GLMs and their statistics (coefficients, *t*‐values, and *p* values) are shown.

**Figure 4:**
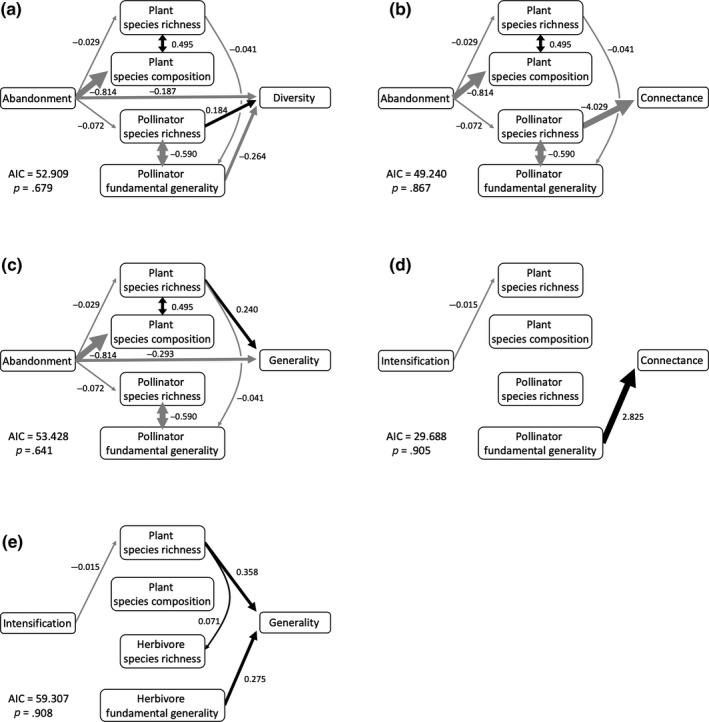
The results of the piecewise structural equation modeling (piecewise *SEM*) for the effects of land‐use changes that were significant on the network structure (see Table 2). The effects of abandonment on pollination network (a) diversity, (b) connectance, (c) generality, and the effects of intensification on (d) pollination network connectance, and (e) herbivory network generality were examined. Illustrated models are the best models and all the paths are significant or marginally significant (*p* < .1). Black and gray arrows indicate positive and negative effects with their width representing the standardized path coefficients. For easy visualization, the effects of season as covariates are not shown. Model fits are shown as *p* values and AIC in the lower‐left corner, and all the models fitted the data well (*p* > .05)

The piecewise *SEM* models showed that land‐use changes affected the network indices in variable ways depending on the interaction types and land‐use change types (Figure 4). The effects of abandonment on connectance, diversity, and generality of pollination networks were mediated by both network size and species composition, and a part of the effects was independent of either. On the contrary, the only path remained in the best model of the effect of intensification on herbivory generality was via changes in plant species richness. The best model of the effect of intensification on connectance of pollination networks did not indicate any significant path tracing from intensification to connectance, possibly because each path was weak and not statistically detectable by this analysis.

## 4 DISCUSSION

The present study demonstrated that either direction of land‐use changes (abandonment or intensification) result in decreased plant and insect species richness and alter their composition, which in turn modify their interaction network structures. Notably, we report the contrasting consequences of land‐use changes between herbivory and pollination networks; abandonment reduced the generality of pollination networks, whereas intensification reduced the generality of herbivory networks. We further found that the mechanisms underlying these effects were also different between herbivory and pollination networks. Though the effects of abandonment on pollination networks were mediated by both changes in network size and species composition and a part of the effects was independent of either changes, the effect of intensification on herbivory networks was mediated only by plant species richness.

The question of whether land‐use changes have correlated effects on multiple interaction networks has been poorly investigated, and only a couple of studies have investigated several types of interaction networks in parallel (Albrecht et al., 2014; Grass et al., 2018). Though our finding is consistent with an earlier study showing that habitat modification affected antagonistic and mutualistic networks in different ways (Grass et al., 2018), it is distinct from the earlier one in that we found the effects of bidirectional land‐use changes were contrasting between herbivory and pollination networks. The uneven effects on antagonistic and mutualistic interaction networks can result in the variable mixtures of different interaction types, which has been theoretically expected to affect ecological and evolutional dynamics of communities (Fontaine et al., 2011; Mougi & Kondoh, 2012). Therefore, land‐use changes can affect ecological and evolutionary dynamics in the long run, as well as the present community structure. In addition, such different responses of multiple types of interaction networks against land‐use changes indicate that consideration of only a single type of interaction may be insufficient to assess the total impact of land‐use changes on multitaxonomic communities (Pocock et al., 2012).

### 4.1 Patterns and mechanisms of the effects on pollination networks

We found that pollination networks in abandoned fields were less diverse and less generalized than those in extensively managed fields. Despite the recent recognition of land‐use abandonment as a threat to terrestrial biodiversity (Koshida & Katayama, 2018; Middleton, 2013), most studies investigating interaction networks have focused only on land‐use intensification (e.g., Baldock et al., 2015; Marrero et al., 2014; Theodorou et al., 2017; Vanbergen et al., 2014). Nevertheless, our findings are in agreement with an earlier study showing that pollination networks in hardly grazed habitats were less diverse (Lázaro et al., 2016).

Our *SEM* analyses revealed that the effects of abandonment on pollination networks were attributed to both network size and species composition. As the network indices are related to network size (Bersier et al., 1999; Fründ et al., 2016; Goldwasser & Roughgarden, 1997), the significant reduction in plant and pollinator species richness in abandoned fields resulted in lower generality and diversity of pollination networks, respectively. We also found that network diversity was affected by pollinator generalist–specialist ratio, and the ratio was determined by plant species richness. In abandoned fields, decrease in plant species richness led to the lower proportion of specialist pollinator species, probably due to their sensitivity to plant species loss (Weiner et al., 2014). In such generalist‐biased networks, the pollination interactions were dominated by a few generalist pollinator species and the network structure could be less diverse. Additionally, the *SEM* analyses indicated the significant effect of abandonment on pollination networks independent of network size or species composition. This effect can be explained by the plastic changes in flowering of some plant species. In abandoned fields where mowing had ceased, a few perennial plant species (*Pueraria lobata* and *Miscanthus sinensis*) have grown to tall vegetation and some plant species characteristic to grasslands exhibit reduced flowering in that shaded environment (Brys, Jacquemyn, Endels, Blust, & Hermy, 2004; Lindborg, Cousins, & Eriksson, 2005). For example, *Justicia procumbens*, one of the common grassland species, occurred in four extensively managed plots and three abandoned plots in our study. Although we found the flowers in two of the extensively managed plots, no flowering individuals were found in the abandoned plots. Such plastic changes in flowering trait of plant species could not be detected by looking at the total species richness or composition, but probably affected the pollination interaction network structures.

### 4.2 Pattern and mechanism of the effects on herbivory networks

The herbivory networks were less generalized in intensified fields than in extensively managed fields. This finding agrees with the previous meta‐analysis by de Araújo et al. (2015) that showed the positive effect of land‐use intensity on herbivory network specialization. The *SEM* indicates that the lower generality of herbivory networks was attributed to reduction in plant species richness in the intensified fields. Even though intensification did not alter the plant and herbivore community composition, the reduction in total plant species richness limited the number of available host plant species and decreased the network generality (de Araújo et al., 2015). Interestingly, though we found that the lower generality in intensified fields was attributed to the herbivore generalist–specialist ratio (fundamental generality), the ratio was not related to the reduction in plant species richness contrary to the expectation (Weiner et al., 2014). The results indicate that the reduction in plant species richness in intensified fields should have limited the host availability for some generalist herbivore species, but it was not so much to cause secondary extinction of specialist species. Therefore, if the land‐use change was more intense and host plant species of some specialist species were lost, we would find that the lower plant species richness leads to higher generalist–specialist ratio which in turn positively affects the network generality.

## 5 CONCLUSION

The importance of conservation of interaction networks as well as species diversity has been stressed (Tylianakis et al., 2010) and numerous previous studies have evaluated the effects of land‐use changes focusing on a single type of interaction network (Lázaro et al., 2016; Tylianakis et al., 2007). Our study demonstrated that herbivory and pollination networks respond differently to land‐use intensification and abandonment, which indicates the importance of considering multiple types of interaction networks in parallel.

Our study showed that extensive management can maintain plant–insect interaction network diversity and generality, which is critical for community dynamics and stability (Bascompte et al., 2006; Thébault & Fontaine, 2010). For example, a higher generality of pollinator and herbivore species in extensively managed fields indicates the high resource redundancy that stabilizes the community dynamics and prevents secondary extinction (Brodie et al., 2014; McCann, Hastings, & Huxel, 1998). Therefore, the present results suggest the possible, and underappreciated, long‐term benefit of maintaining traditional extensive management practices.

## CONFLICT OF INTEREST

None declared.

## AUTHORS' CONTRIBUTIONS

N. S., K. U., and T. Y. conceived the study. N. S. and K. U. conducted fieldwork. N. S. analyzed the data. N. S. led the writing of the manuscript. All authors actively contributed to the manuscript's results and discussion and approved the final manuscript for publication.

## Data Availability

The data used to support the findings of this study are archived in figshare repository: https://doi.org/10.6084/m9.figshare.10000352.v1
