# Supplementary figures and images for: Contrasting effects of land‐use changes on herbivory and pollination networks
Source: Ecol Evol. 2019 Nov 20;9(23):13585–95. doi: 10.1002/ece3.5814 (PMC6912900; doi:10.1002/ece3.5814)

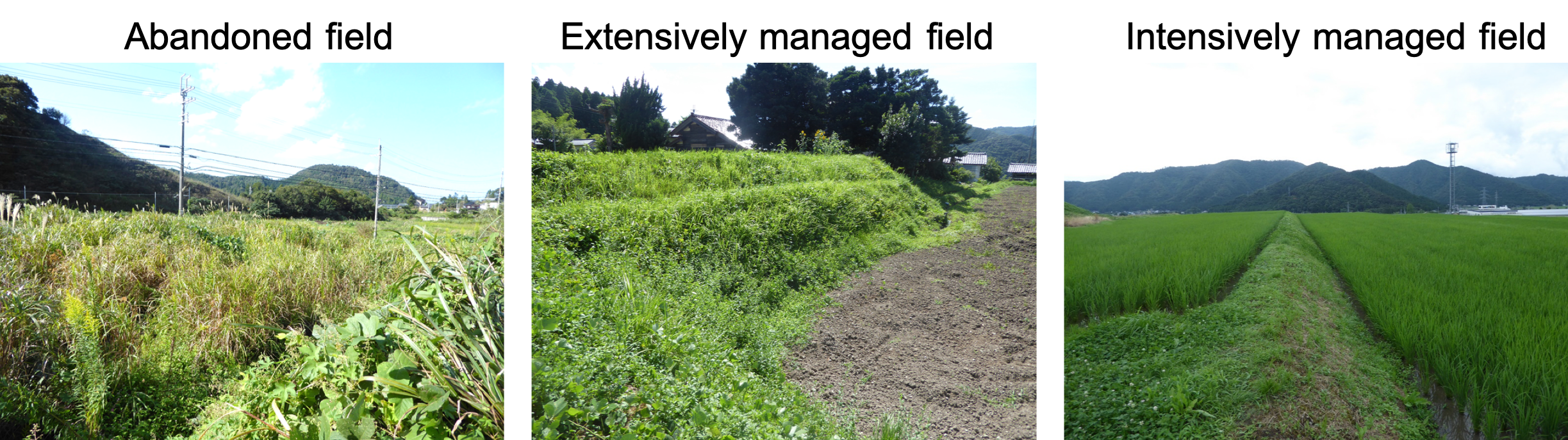

Supplement: Supplementary file 1 [file ECE3-9-13585-s001.png]

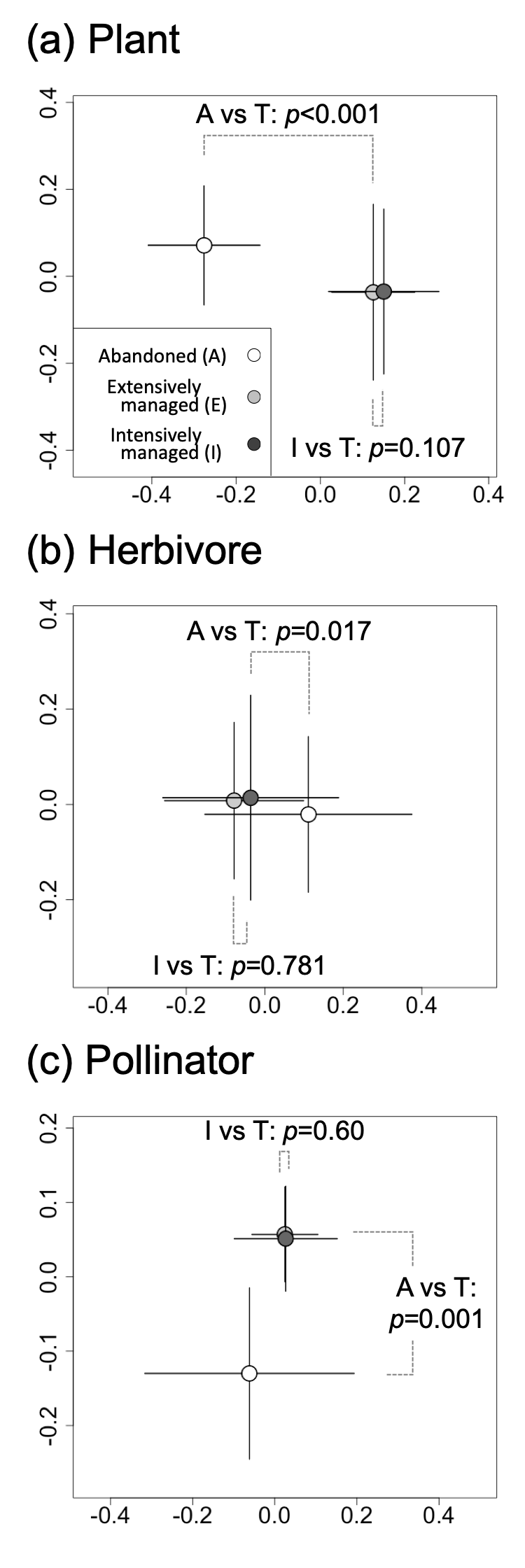

Supplement: Supplementary file 2 [file ECE3-9-13585-s002.png]
