# Supplementary material for: Contrasting effects of land‐use changes on herbivory and pollination networks
Source: Ecol Evol. 2019 Nov 20;9(23):13585–95. doi: 10.1002/ece3.5814 (PMC6912900; doi:10.1002/ece3.5814)
Supplement: Supplementary file 3 [file ECE3-9-13585-s003.docx]

**Figure S1**. Photographs of representative plots for the different land-use types.

**Figure S2**. Effects of land-use changes (abandonment or intensification) on plant, herbivore, and pollinator species composition. Means ± SDs of the first and second NMDS axes for each land-use type are illustrated. Comparisons were performed using perMANOVA and the significance levels (*P*-values) of the differences are shown. The stress values, which represent fitting of the ordination to the observed data, were 0.206, 0.149, and 0.158 for the plant, herbivore, and pollinator communities, respectively.

Table S1. List of insect and plant species observed in this study with the species IDs used in Figures 2 and S3.

Insect Species

| ID | Scientific Name | Order | Family |
| --- | --- | --- | --- |
| 1 | *Chrysomelidae* sp*.* | Coleoptera | Chrysomelidae |
| 2 | *Eumolpinae* sp*.* | Coleoptera | Chrysomelidae |
| 3 | *Altica caerulescens* | Coleoptera | Chrysomelidae |
| 4 | *Altica oleracea* | Coleoptera | Chrysomelidae |
| 5 | *Atrachya menetriesi* | Coleoptera | Chrysomelidae |
| 6 | *Aulacophora indica* | Coleoptera | Chrysomelidae |
| 7 | *Basilepta fulvipes* | Coleoptera | Chrysomelidae |
| 8 | *Chrysolina aurichalcea* | Coleoptera | Chrysomelidae |
| 9 | *Galerucella grisescens* | Coleoptera | Chrysomelidae |
| 10 | *Lema coronata* | Coleoptera | Chrysomelidae |
| 11 | *Lema diversa* | Coleoptera | Chrysomelidae |
| 12 | *Lilioceris merdigera* | Coleoptera | Chrysomelidae |
| 13 | *Monolepta dichroa* | Coleoptera | Chrysomelidae |
| 14 | *Episomus turritus* | Coleoptera | Curculionidae |
| 15 | *Eugnathus distinctus* | Coleoptera | Curculionidae |
| 16 | *Lixus acutipennis* | Coleoptera | Curculionidae |
| 17 | *Lixus depressipennis* | Coleoptera | Curculionidae |
| 18 | *Lixus impressiventris* | Coleoptera | Curculionidae |
| 19 | *Sternuchopsis trifidus* | Coleoptera | Curculionidae |
| 20 | *Ampedus japonicus* | Coleoptera | Elateridae |
| 21 | *Anomala albopilosa* | Coleoptera | Scarabaeidae |
| 22 | *Exomala orientalis* | Coleoptera | Scarabaeidae |
| 23 | *Gametis jucunda* | Coleoptera | Scarabaeidae |
| 24 | *Maladera japonica* | Coleoptera | Scarabaeidae |
| 25 | *Mimela splendens* | Coleoptera | Scarabaeidae |
| 26 | *Popillia japonica* | Coleoptera | Scarabaeidae |
| 27 | *Staphylinidae* sp*.* | Coleoptera | Staphylinidae |
| 28 | *Diptera* sp*.* | Diptera | - |
| 29 | *Diptera* sp*.*1 | Diptera | - |
| 30 | *Anthomyiidae* sp*.* | Diptera | Anthomyiidae |
| 31 | *Stomorhina obsoleta* | Diptera | Calliphoridae |
| 32 | *Aspidomorpha difformis* | Diptera | Chrysomelidae |
| 33 | *Cassida piperata* | Diptera | Chrysomelidae |
| 34 | *Heleomyzidae* sp*.* | Diptera | Heleomyzidae |
| 35 | *Muscidae* sp*.* | Diptera | Muscidae |
| 36 | *Musca hervei* | Diptera | Muscidae |
| 37 | *Sarcophagidae* sp*.* | Diptera | Sarcophagidae |
| 38 | *Odontomyia garatas* | Diptera | Stratiomyidae |
| 39 | *Syrphidae* sp*.* | Diptera | Syrphidae |
| 40 | *Episyrphus balteatus* | Diptera | Syrphidae |
| 41 | *Eristalinus quinquestriatus* | Diptera | Syrphidae |
| 42 | *Eristalis cerealis* | Diptera | Syrphidae |
| 43 | *Eristalis tenax* | Diptera | Syrphidae |
| 44 | *Melanostoma mellinum* | Diptera | Syrphidae |
| 45 | *Melanostoma orientale* | Diptera | Syrphidae |
| 46 | *Mesembrius flaviceps* | Diptera | Syrphidae |
| 47 | *Phytomia zonata* | Diptera | Syrphidae |
| 48 | *Sphaerophoria macrogaster* | Diptera | Syrphidae |
| 49 | *Tachinidae* sp*.* | Diptera | Tachinidae |
| 50 | *Gymnosoma rotundata* | Diptera | Tachinidae |
| 51 | *Prosena siberita Fabricius* | Diptera | Tachinidae |
| 52 | *Heteroptera* sp*.* | Hemiptera | - |
| 53 | *Aphidoidea* | Hemiptera | - |
| 54 | *Aphidoidea* sp.1 | Hemiptera | - |
| 55 | *Aphidoidea* sp.2 | Hemiptera | - |
| 56 | *Leptocorisa chinensis* | Hemiptera | Alydidae |
| 57 | *Riptortus linearis* | Hemiptera | Alydidae |
| 58 | *Aphis craccivora* | Hemiptera | Aphididae |
| 59 | *Macrosiphoniella yomogicola* | Hemiptera | Aphididae |
| 60 | *Megoura crassicauda* | Hemiptera | Aphididae |
| 61 | *Melanaphis japonica* | Hemiptera | Aphididae |
| 62 | *Uroleucon nigrotuberculatum* | Hemiptera | Aphididae |
| 63 | *Uroleucon picridis* | Hemiptera | Aphididae |
| 64 | *Cletus punctiger* | Hemiptera | Coreidae |
| 65 | *Cletus schmidti* | Hemiptera | Coreidae |
| 66 | *Homoeocerus striicornis* | Hemiptera | Coreidae |
| 67 | *Homoeocerus unipunctatus* | Hemiptera | Coreidae |
| 68 | *Hygia opaca* | Hemiptera | Coreidae |
| 69 | *Riptortus pedestris* | Hemiptera | Coreidae |
| 70 | *Nysius plebeius* | Hemiptera | Lygaeidae |
| 71 | *Charagochilus angusticollis* | Hemiptera | Miridae |
| 72 | *Lygocoris lucorum* | Hemiptera | Miridae |
| 73 | *Stenotus rubrovittatus* | Hemiptera | Miridae |
| 74 | *Dolycoris baccarum* | Hemiptera | Pentatomidae |
| 75 | *Eurydema rugosa* | Hemiptera | Pentatomidae |
| 76 | *Eysarcoris ventralis* | Hemiptera | Pentatomidae |
| 77 | *Halyomorpha halys* | Hemiptera | Pentatomidae |
| 78 | *Nezara antennata* | Hemiptera | Pentatomidae |
| 79 | *Piezodorus hybneri* | Hemiptera | Pentatomidae |
| 80 | *Coptosoma biguttulum* | Hemiptera | Plataspidae |
| 81 | *Coptosoma parvipictum* | Hemiptera | Plataspidae |
| 82 | *Rhopalus maculatus* | Hemiptera | Rhopalidae |
| 83 | *Hymenoptera* sp*.* | Hymenoptera | - |
| 84 | *Symphyta* sp*.* | Hymenoptera | - |
| 85 | *Apoidea* sp*.* | Hymenoptera | - |
| 86 | *Apoidea* sp*.* 1 | Hymenoptera | - |
| 87 | *Andrenidae* sp*.* | Hymenoptera | Andrenidae |
| 88 | *Andrenidae* sp*.* 1 | Hymenoptera | Andrenidae |
| 89 | *Andrenidae* sp*.* 2 | Hymenoptera | Andrenidae |
| 90 | *Apis* sp*.* | Hymenoptera | Apidae |
| 91 | *Bombus* sp*.* | Hymenoptera | Apidae |
| 92 | *Ceratina flavipes* | Hymenoptera | Apidae |
| 93 | *Xylocopa appendiculata* | Hymenoptera | Apidae |
| 94 | *Eumenes micado* | Hymenoptera | Eumenidae |
| 95 | *Formicidae* sp*.* | Hymenoptera | Formicidae |
| 96 | *Halictidae* sp*.* | Hymenoptera | Halictidae |
| 97 | *Campsomeris prismatica* | Hymenoptera | Scoliidae |
| 98 | *Scolia oculata* | Hymenoptera | Scoliidae |
| 99 | *Allantus luctifer* | Hymenoptera | Tenthredinidae |
| 100 | *Allantus nigrocaeruleus* | Hymenoptera | Tenthredinidae |
| 101 | *Dolerus subfasciatus* | Hymenoptera | Tenthredinidae |
| 102 | *Lepidoptera* sp*.* | Lepidoptera | - |
| 103 | *Arctiidae* sp*.* | Lepidoptera | Arctiidae |
| 104 | *Geometridae* sp*.* | Lepidoptera | Geometridae |
| 105 | *Ascotis selenaria* | Lepidoptera | Geometridae |
| 106 | *Pylargosceles steganioides steganioides* | Lepidoptera | Geometridae |
| 107 | *Parnara guttata* | Lepidoptera | Hesperiidae |
| 108 | *Parasa lepida* | Lepidoptera | Limacodidae |
| 109 | *Euchrysops cnejus* | Lepidoptera | Lycaenidae |
| 110 | *Everes argiades* | Lepidoptera | Lycaenidae |
| 111 | *Lycaena phlaeas* | Lepidoptera | Lycaenidae |
| 112 | *Pseudozizeeria maha* | Lepidoptera | Lycaenidae |
| 113 | *Lymantriinae* sp*.* | Lepidoptera | Lymantriidae |
| 114 | *Autographa gamma* | Lepidoptera | Noctuidae |
| 115 | *Cucullia fraterna* | Lepidoptera | Noctuidae |
| 116 | *Cucullia kurilullia* | Lepidoptera | Noctuidae |
| 117 | *Sarcopolia illoba* | Lepidoptera | Noctuidae |
| 118 | *Thysanoplusia intermixta* | Lepidoptera | Noctuidae |
| 119 | *Viminia rumicis* | Lepidoptera | Noctuidae |
| 120 | *Cynthia cardui* | Lepidoptera | Nymphalidae |
| 121 | *Papilio machaon* | Lepidoptera | Papilionidae |
| 122 | *Colias erate* | Lepidoptera | Pieridae |
| 123 | *Eurema mandarina* | Lepidoptera | Pieridae |
| 124 | *Pieris melete* | Lepidoptera | Pieridae |
| 125 | *Pieris rapae* | Lepidoptera | Pieridae |
| 126 | *Psychidae* sp*.* | Lepidoptera | Psychidae |
| 127 | *Bocchoris inspersalis* | Lepidoptera | Pyralidae |
| 128 | *Spoladea recurvalis* | Lepidoptera | Pyralidae |
| 129 | *Sphingidae* sp*.* | Lepidoptera | Sphingidae |
| 130 | *Cephonodes hylas* | Lepidoptera | Sphingidae |
| 131 | *Clanis bilineata* | Lepidoptera | Sphingidae |
| 132 | *Oxya yezoensis* | Orthoptera | Acrididae |
| 133 | *Parapodisma setouchiensis* | Orthoptera | Acrididae |
| 134 | *Patanga japonica* | Orthoptera | Acrididae |
| 135 | *Stethophyma magister* | Orthoptera | Acrididae |
| 136 | *Pteronemobius ohmachii* | Orthoptera | Gryllidae |
| 137 | *Teleogryllus emma* | Orthoptera | Gryllidae |
| 138 | *Atractomorpha lata* | Orthoptera | Pyrgomorphidae |
| 139 | *Euparatettix insularis* | Orthoptera | Tetrigidae |
| 140 | *Conocephalus chinensis* | Orthoptera | Tettigoniidae |
| 141 | *Conocephalus melaenus* | Orthoptera | Tettigoniidae |
| 142 | *Ducetia japonica* | Orthoptera | Tettigoniidae |
| 143 | *Eobiana engelhardti* | Orthoptera | Tettigoniidae |
| 144 | *Phaneroptera falcata* | Orthoptera | Tettigoniidae |

Plant Species

| ID | Scientific Name | Order | Family |
| --- | --- | --- | --- |
| 1 | *Sagittaria trifolia* L. | Alismatales | Alismataceae |
| 2 | *Pinellia ternata* | Alismatales | Araceae |
| 3 | *Hydrocotyle ramiflora* | Apiales | Umbelliferae |
| 4 | *Hydrocotyle sibthorpioides* | Apiales | Umbelliferae |
| 5 | *Oenanthe javanica* | Apiales | Umbelliferae |
| 6 | *Spiranthes sinensis* var*. amoena* | Asparagales | Orchidaceae |
| 7 | *Hemerocallis fulva* var*. kwanso* | Asparagales | Xanthorrhoeaceae |
| 8 | *Artemisia indica* var*. maximowiczii* | Asterales | Compositae |
| 9 | *Aster yomena* | Asterales | Compositae |
| 10 | *Cirsium japonicum* | Asterales | Compositae |
| 11 | *Compositae* sp*.* | Asterales | Compositae |
| 12 | *Conyza canadensis* | Asterales | Compositae |
| 13 | *Conyza sumatrensis* | Asterales | Compositae |
| 14 | *Eclipta thermalis* | Asterales | Compositae |
| 15 | *Erigeron annuus* | Asterales | Compositae |
| 16 | *Gnaphalium japonicum* | Asterales | Compositae |
| 17 | *Inula britannica* subsp*. japonica* | Asterales | Compositae |
| 18 | *Ixeridium dentatum* | Asterales | Compositae |
| 19 | *Ixeris japonica* | Asterales | Compositae |
| 20 | *Lactuca indica* | Asterales | Compositae |
| 21 | *Leucanthemum* Mill*.* | Asterales | Compositae |
| 22 | *Petasites japonicus* | Asterales | Compositae |
| 23 | *Picris hieracioides* subsp*. japonica* | Asterales | Compositae |
| 24 | *Solidago altissima* | Asterales | Compositae |
| 25 | *Sonchus asper* | Asterales | Compositae |
| 26 | *Sonchus oleraceus* | Asterales | Compositae |
| 27 | *Taraxacum japonicum* Koidz*.* | Asterales | Compositae |
| 28 | *Rorippa palustris* | Brassicales | Cruciferae |
| 29 | *Achyranthes bidentata* var*. japonica* | Caryophyllales | Amaranthaceae |
| 30 | *Cerastium fontanum* subsp*. vulgare* var*. angustifolium* | Caryophyllales | Caryophyllaceae |
| 31 | *Cerastium glomeratum* | Caryophyllales | Caryophyllaceae |
| 32 | *Sagina japonica* | Caryophyllales | Caryophyllaceae |
| 33 | *Stellaria aquatica* | Caryophyllales | Caryophyllaceae |
| 34 | *Stellaria media* | Caryophyllales | Caryophyllaceae |
| 35 | *Fallopia japonica* | Caryophyllales | Polygonaceae |
| 36 | *Persicaria hydropiper* | Caryophyllales | Polygonaceae |
| 37 | *Persicaria longiseta* | Caryophyllales | Polygonaceae |
| 38 | *Persicaria thunbergii* | Caryophyllales | Polygonaceae |
| 39 | *Rumex acetosa* | Caryophyllales | Polygonaceae |
| 40 | *Rumex japonicus* Houtt*.* | Caryophyllales | Polygonaceae |
| 41 | *Portulaca oleracea* | Caryophyllales | Portulacaceae |
| 42 | *Commelina communis* | Commelinales | Commelinaceae |
| 43 | *Murdannia keisak* | Commelinales | Commelinaceae |
| 44 | *Deutzia crenata* | Cornales | Saxifragaceae |
| 45 | *Dioscorea japonica* | Dioscoreales | Dioscoreaceae |
| 46 | *Equisetum arvense* | Equisetales | Equisetaceae |
| 47 | *Lysimachia japonica* | Ericales | Primulaceae |
| 48 | *Aeschynomene indica* | Fabales | Leguminosae |
| 49 | *Amphicarpaea bracteata* subsp*. edgeworthii* var*. japonica* | Fabales | Leguminosae |
| 50 | *Desmodium podocarpum* subsp*. oxyphyllum* | Fabales | Leguminosae |
| 51 | *Glycine max* subsp*. soja* | Fabales | Leguminosae |
| 52 | *Kummerowia striata* | Fabales | Leguminosae |
| 53 | *Lespedeza pilosa* | Fabales | Leguminosae |
| 54 | *Medicago lupulina* | Fabales | Leguminosae |
| 55 | *Pueraria lobata* | Fabales | Leguminosae |
| 56 | *Trifolium dubium* | Fabales | Leguminosae |
| 57 | *Trifolium pratense* | Fabales | Leguminosae |
| 58 | *Trifolium repens* | Fabales | Leguminosae |
| 59 | *Vicia sativa* subsp*. nigra* | Fabales | Leguminosae |
| 60 | *Vigna angularis* var*. nipponensis* | Fabales | Leguminosae |
| 61 | *Geranium thunbergii* | Geraniales | Geraniaceae |
| 62 | *Hypericum erectum* | Guttiferales | Guttiferae |
| 63 | *Hypericum laxum* | Guttiferales | Guttiferae |
| 64 | *Juncus decipiens* | Juncales | Juncaceae |
| 65 | *Luzula capitata* | Juncales | Juncaceae |
| 66 | *Justicia procumbens* | Lamiales | Acanthaceae |
| 67 | *Clinopodium chinense* subsp*. grandiflorum* var*. urticifolium* | Lamiales | Labiatae |
| 68 | *Clinopodium gracile* | Lamiales | Labiatae |
| 69 | *Glechoma hederacea* subsp*. grandis* | Lamiales | Labiatae |
| 70 | *Lycopus cavaleriei* | Lamiales | Labiatae |
| 71 | *Mosla dianthera* | Lamiales | Labiatae |
| 72 | *Plantago asiatica* | Lamiales | Plantaginaceae |
| 73 | *Veronica arvensis* | Lamiales | Scrophulariaceae |
| 74 | *Veronica peregrina* | Lamiales | Scrophulariaceae |
| 75 | *Liliaceae.sp* | Liliales | Liliaceae |
| 76 | *Acalypha australis* | Malpighiales | Euphorbiaceae |
| 77 | *Chamaesyce nutans* | Malpighiales | Euphorbiaceae |
| 78 | *Mallotus japonicus* | Malpighiales | Euphorbiaceae |
| 79 | *Viola grypoceras* | Malpighiales | Violaceae |
| 80 | *Viola verecunda* | Malpighiales | Violaceae |
| 81 | *Epilobium pyrricholophum* | Myrtales | Onagraceae |
| 82 | *Ludwigia epilobioides* | Myrtales | Onagraceae |
| 83 | *Oenothera stricta* | Myrtales | Onagraceae |
| 84 | *Oxalis corniculata* | Oxalidales | Oxalidaceae |
| 85 | *Oxalis dillenii* | Oxalidales | Oxalidaceae |
| 86 | *Houttuynia cordata* | Piperales | Saururaceae |
| 87 | *Carex gibba* | Poales | Cyperaceae |
| 88 | *Carex leucochlora* | Poales | Cyperaceae |
| 89 | *Carex* sp*.* | Poales | Cyperaceae |
| 90 | *Cyperus flavidus* | Poales | Cyperaceae |
| 91 | *Agrostis clavata* subsp*. matsumurae* | Poales | Gramineae |
| 92 | *Anthoxanthum odoratum* | Poales | Gramineae |
| 93 | *Arthraxon hispidus* | Poales | Gramineae |
| 94 | *Briza maxima* | Poales | Gramineae |
| 95 | *Briza minor* | Poales | Gramineae |
| 96 | *Cynodon dactylon* | Poales | Gramineae |
| 97 | *Digitaria ciliaris* | Poales | Gramineae |
| 98 | *Echinochloa crus-galli* var*. aristata* | Poales | Gramineae |
| 99 | *Eleusine indica* | Poales | Gramineae |
| 100 | *Elymus tsukushiensis* var*. transiens* | Poales | Gramineae |
| 101 | *Gramineae* sp*.* | Poales | Gramineae |
| 102 | *Imperata cylindrica* var*. koenigii* | Poales | Gramineae |
| 103 | *Lolium multiflorum* | Poales | Gramineae |
| 104 | *Microstegium vimineum* | Poales | Gramineae |
| 105 | *Miscanthus sinensis* | Poales | Gramineae |
| 106 | *Oplismenus undulatifolius* | Poales | Gramineae |
| 107 | *Paspalum thunbergii* | Poales | Gramineae |
| 108 | *Pennisetum alopecuroides* | Poales | Gramineae |
| 109 | *Poa acroleuca* | Poales | Gramineae |
| 110 | *Poa annua* | Poales | Gramineae |
| 111 | *Setaria pumila* | Poales | Gramineae |
| 112 | *Setaria viridis* | Poales | Gramineae |
| 113 | *Trisetum bifidum* | Poales | Gramineae |
| 114 | *Vulpia myuros* | Poales | Gramineae |
| 115 | *Cocculus orbiculatus* | Ranunculales | Menispermaceae |
| 116 | *Ranunculus japonicus* | Ranunculales | Ranunculaceae |
| 117 | *Ranunculus silerifolius* var*. glaber* | Ranunculales | Ranunculaceae |
| 118 | *Thalictrum minus* var*. hypoleucum* | Ranunculales | Ranunculaceae |
| 119 | *Humulus scandens* | Rosales | Moraceae |
| 120 | *Duchesnea chrysantha* | Rosales | Rosaceae |
| 121 | *Potentilla anemonifolia* | Rosales | Rosaceae |
| 122 | *Galium trachyspermum* | Rubiales | Rubiaceae |
| 123 | *Neanotis hirsuta* | Rubiales | Rubiaceae |
| 124 | *Paederia scandens* | Rubiales | Rubiaceae |
| 125 | *Sedum bulbiferum* | Saxifragales | Crassulaceae |
| 126 | *Calystegia　japonica* | Solanales | Convolvulaceae |
| 127 | *Boehmeria japonica* var*. longispica* | Urticales | Urticaceae |
| 128 | *Boehmeria nivea* var*. nipononivea* | Urticales | Urticaceae |
| 129 | *Ampelopsis glandulosa* var*. heterophylla* | Vitales | Vitaceae |
| 130 | *Cayratia japonica* | Vitales | Vitaceae |
| 131 | *Pteridopsida* sp*.* | - | - |
